# The Duration of Negative Pressure Wound Therapy Can Be Reduced Using the HeartShield Device in Patients With Deep Sternal Wound Infection

**Published:** 2014-04-03

**Authors:** Richard Ingemansson, Malin Malmsjö, Sandra Lindstedt

**Affiliations:** ^a^Department of Cardiothoracic Surgery, Lund University and Skåne University Hospital, Lund, Sweden; ^b^Department of Ophthalmology, Lund University and Skåne University Hospital, Lund, Sweden

**Keywords:** Wound, heart, sternotomy, NPWT, vacuum

## Abstract

**Background:** Heart rupture resulting in lethal bleeding is a devastating complication associated with negative pressure wound therapy (NPWT) in patients with deep sternal wound infection (DSWI). We have previously reported that the use of a protective HeartShield device in combination with NPWT decreases the risk of damage to the heart. This article presents a retrospective analysis of NPWT duration with and without the HeartShield device. **Subjects and patients:** The study included 6 patients treated with the HeartShield device in combination with NPWT and 6 patients treated with conventional NPWT during the same time period. The duration of active treatment time was measured. **Results:** The median duration of NPWT was 8 days (range: 6-14 days) in the HeartShield device NPWT group and 14 days in the conventional group (range: 10-18 days). The difference was statistically significant (*P* < .05). **Conclusions:** It appears that the treatment of patients with DSWI with the HeartShield device reduces the duration of NPWT.

The use of negative pressure wound therapy (NWPT) has gained acceptance in the treatment of deep sternal wound infection (DSWI) because of excellent clinical results and significantly lower mortality and morbidity rates, and it is today the standard mode of treatment at many cardiac surgery centers throughout the world.^1^^-^8 However, a serious complication associated with the use of NPWT is right ventricular rupture; the incidence being 4% to 7% of patients treated for DSWI with NPWT following cardiac surgery.^9^^,^^10^ Furthermore, the Federal Drug Administration has published 2 reports in which they discuss the issue of bleeding during NPWT in the mediastinum.^11^^,^^12^

We have previously described the cause of right ventricular rupture using magnetic resonance imaging to elucidate the problem.^13^^-^15 The heart was shown to be drawn up toward the thoracic wall; the right ventricle bulged into the space between the sternal edges, and the sharp edges of the sternum protruded into the anterior surface of the heart, in some cases resulting in damage to the right ventricle. However, we found that these events could be prevented by inserting a protective device between the anterior part of the heart and the inside of the thoracic wall. Our group has performed extensive experiments on pigs, and 4 papers have been published demonstrating improved drainage, good wound stability, and less marks and bleeding of the heart and lungs, without impairing hemodynamics, when using such the HeartShield device.^16^^-^19 We have also presented the application technique and early experience with the Heartshield device in patients.^20^ The use HeartShield device in combination with NPWT has been shown to decrease the risk of damage to the heart in patients.^21^ In the same group of patients, C-reactive protein levels and leukocyte counts dropped faster and bacterial clearance occurred earlier in the HeartShield group compared to the conventional NPWT group.^22^ We suggest that the reason is improved drainage with the device as demonstrated in animal studies^18^ In this study, we compare the duration of NPWT treatment with and without the HeartShield device.

## SUBJECTS AND METHODS

### Patients

Between October 2011 and April 2012, 12 patients were treated with NPWT because of DSWI following cardiac surgery. Six of these patients were treated with conventional NPWT and 6 with NPWT using the HeartShield device (Shieldheart Medtech AB, Lund, Sweden). These patients have been included in previous studies for describing the method,^20^ signs of heart damage,^21^ and changes in C-reactive protein levels.^22^ This study was to evaluate the duration of NPWT. The study was approved by the Ethics Committee of Lund University. Adult patients with DSWI after open heart surgery due to cardiac disease were included in the study. Patients with malformations of the thoracic cavity (eg, pectus excavatum or carinatum), patients with a body weight less than 45 kg, or patients with pregnancy were excluded in the study. After the patients had been included, they were randomized to be treated with NPWT only (conventional) and NPWT using HeartShield.

### Conventional NPWT group

Four patients were infected with coagulase-negative staphylococci, 1 with *Staphylococcus aureus* and 1 with *Enterococci Coli*. The mean age of the patients was 71 years (67-80). One patient had insulin-dependent diabetes mellitus; 1 had body mass index higher than 30 kg/m^2^, 1 had chronic obstructive lung disease, and 1 had creatinine level higher than 140. The wound was opened and 5 samples were collected for tissue cultures. A full revision was performed and the wound was rinsed. Three layers of Jelonet (Smith & Nephew, St Petersburg, Florida) were placed over the heart. A layer of foam (KCI, San Antonio, Texas) was placed between the sternal edges, and a second layer was cut and placed presternally. This layer was secured to the surrounding skin by continuous suturing with Dermalon 2:0 (Covidien, Mansfield, Massachusetts). A plastic drape (KCI, San Antonio, Texas) was then applied over the outer foam layer. Holes were made in the plastic drape with a knife, and 2 Trackpads (KCI, San Antonio, Texas) were applied over the drainage holes. These were connected to a negative pressure source using Y-connectors (both from KCI, San Antonio, Texas). A continuous negative pressure of −125 mm Hg was applied.

### The HeartShield device

**Figure 1:**
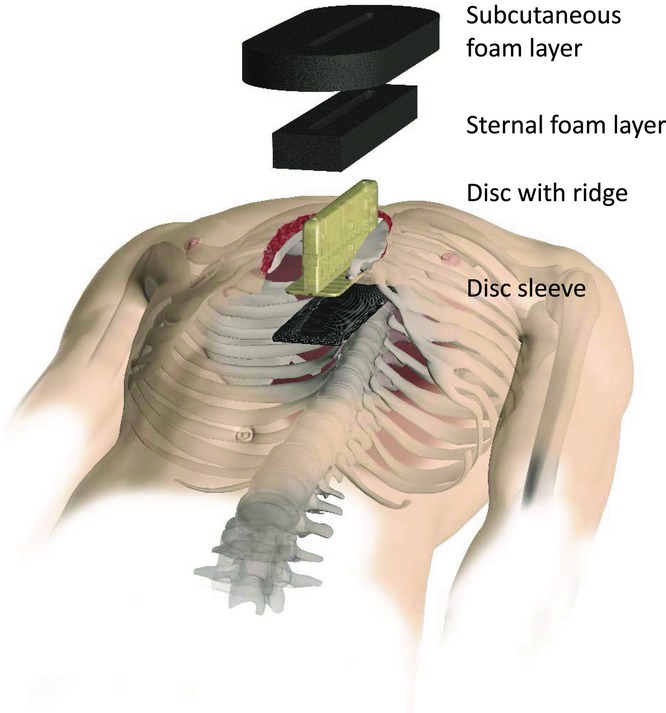
Schematic illustration showing the assembly of the different components of the HeartShield device. The HeartShield device that is intended to protect the heart, lungs, and cardiovascular structures and to shorten the time to closure of sternotomy wounds receiving NPWT. The protective properties are achieved by hindering the heart and lungs from being pulled into the sternum bone edges by the suction force of the negative pressure. The HeartShield device includes the following: The HeartShield disc with ridge, which is a rigid barrier disc, for hindering the heart from being sucked up towards the sternum bone edges by the negative pressure, with a ridge, for ensuring positioning of the device and facilitating negative pressure transduction and drainage through the device. The HeartShield disc sleeve, which is made of foam that is covered with a low-adherence wound contact layer, for providing smooth surface to the heart. The wound contact layer (WCL) hinders ingrowth of tissues or organs into the foam. The WCL is perforated to allow negative pressure transduction and drainage through it. The HeartShield wound filler material, which is an open pore structure foam for transmitting negative pressure to the wound and facilitating drainage, comprising the HeartShield sternal foam layer and the HeartShield subcutaneous foam layer.

The HeartShield device is primarily a protection device designed to prevent cardiac bleeding during NPWT of the mediastinum. It consists of plastic and has a T-shaped cross-section. The base of the HeartShield device is covered with a foam sleeve, and the HeartShield device is inserted into the wound so that the base covers the heart. The central part of the device then lies between the sharp edges of the sternum and protrudes above the level of the skin. Holes in the device allow fluid to be drained from the wound. A schematic illustration showing the assembly of the different components of the HeartShield device can be seen in Fig 1.

### The HeartShield device NPWT group

**Figure 2:**
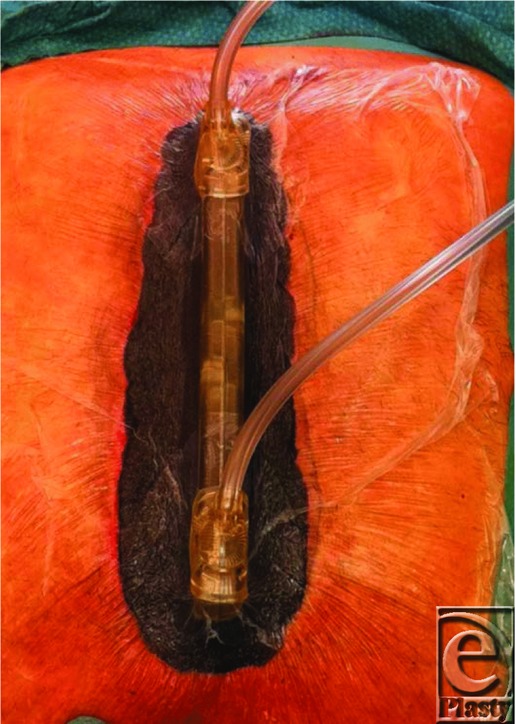
The HeartShield device in the sternotomy wound. The plastic drape has been applied and the drains are in place, exiting the top of the device.

One patient was infected with coagulase-negative staphylococci, 3 with *Staphylococcus aureus*, 1 with *Klebsiella pneumonia*, and 1 with *Propionibacterium acne*. The mean age of the patients was 71 (69-77) years. One patient had insulin-dependent diabetes mellitus; 1 had body mass index higher than 30 kg/m^2^, 2 patients had chronic obstructive lung disease, and 1 had creatinine level higher than 140. The wound was opened, 5 samples were taken for tissue cultures, a full revision was performed, and the wound was rinsed as described earlier. RENASYS-F foam was used (Smith & Nephew, St Petersburg, Florida). A foam sleeve was fitted to the base of the HeartShield device. Two layers of Delnet (Delstar Technologies Inc, Delaware) were cut and laid and sutured onto the foam sleeve to hinder the foam from coming into direct contact with the heart. The HeartShield device was then placed in the wound so that one fourth of the device was above the top of the diaphragm, thus covering the heart and lungs, as well as any graft. A second layer of foam was inserted on either side of the device between the vertical part of the device and the sternal edges to help stabilize the device. A third layer of foam, with the same length as the device, was then placed over the device. This final layer of foam was secured to the surrounding skin by continuous suturing. The wound was closed with an adhesive drape. Two RENASYS Ports (Smith & Nephew, St Petersburg, Florida) were connected to the drainage holes as described earlier (Fig 2). These were connected to a negative pressure source using Y-connectors (Smith & Nephew, St Petersburg, Florida). A continuous negative pressure of −120 mm Hg was applied using RENASYS EZ (Smith & Nephew, St Petersburg, Florida).

### Statistical analysis

Calculations and statistical analyses were performed using GraphPad 4.0 software. Statistical analysis was performed using the Mann-Whitney U test. Statistical significance was defined as *P* < .05 (significant, *), and *P* > .05 (not significant, NS).

## RESULTS

### The duration of NPWT

**Figure 3:**
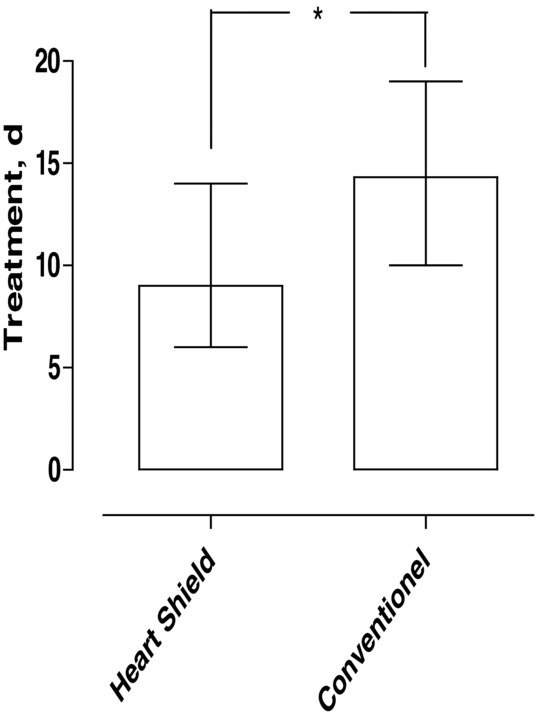
Duration of NPWT in the HeartShield device NPWT group and in the conventional NPWT group (*P* < .05).

The median duration of NPWT in the HeartShield device NPWT group was 8 days (range: 6-14 days), and in the conventional NPWT group, it was 14 days (range: 10-18 days). The difference between the 2 groups was statistically significant (*P* < .05, Fig 3).

### Blood samples

Routine blood samples for hematology, kidney, electrolytes, liver, and pancreas were taken during the whole treatment period. Routine blood samples were normal in all patients when the treatment was finalized.

### Wound edge and macroscopic examination

The site of application of the HeartShield device (the sternotomy wound edges and the underlying heart) and intrathoracic structures and organs are always routinely examined at every occasion. There was no sign of local toxicity. Furthermore, there was no erythema, eschar, edema, or hematoma formation in the group treated with HeartShield device.

### Adverse events

No adverse effects as bleedings from adherences or from the surgical site per se or impaired drainage or impaired cardiac output could be seen during the treatment with the HeartShield device. No injury could be seen on the right or the left lung during the treatment with the HeartShield device. The device was properly positioned during the treatment such that it became stable and did not drift. No ingrowth into the device could be observed during the treatment with the HeartShield device. No other adverse event was seen.

All 12 patients in the study survived the treatment. No adverse events in terms of damage to the heart, lungs, or grafts or major bleeding were observed during the treatment period.

## DISCUSSION

The use of NWPT has gained acceptance in the treatment of DSWI, and it is today the standard mode of treatment at many cardiac surgery centers throughout the world. However, there are increasing reports of deaths and serious complications associated with the use of NPWT, of which right ventricular heart rupture is the most frequent.^9^^,^^10^,^23^^-^25 The use of a rigid barrier has been suggested to offer protection against this lethal complication by preventing the heart from being drawn up against the sharp edges of the sternum.^14^^,^^15^

We have investigated the HeartShield device extensively on pigs and were able to demonstrate less epicardial petechial bleeding of the heart and lungs upon completion of therapy, indicating the absence of contact between the heart and the rough sternal edges, and thus a reduced risk of organ rupture.^19^ The same observations were later made in patients treated for DSWI with the HeartShield device protection device.^21^ In that study, we could demonstrate an all-or-none effect regarding epicardial petechial bleedings with or without the protective device. In another pig study, we were able to demonstrate better wound drainage when using the HeartShield device.^14^ In that study, the device not only provided excellent drainage of the pericardium but also allowed drainage of the pleura. Drainage of the pleura was not possible in wounds treated with conventional NPWT.^18^ The improved drainage is probably due to improvements in the drainage properties of the foam. By placing the device substernal, the foam will extend deeper into the wound, thereby increasing the drainage capacity. The area of foam over which the negative pressure extends will increase because the base of the device will prevent the foam from contracting, thereby increasing the foam-wound contact area. Also, the base of the device helps to hold out the foam laterally, so that it comes closer to the pleura, facilitating drainage. The pressure gradient in the foam, from the bottom up to the drain can sometimes be substantial, especially in wounds with high fluid secretion. Because of the drainage holes in the device, fluid will never have to be transported more than 1 to 2 cm through the foam before it can be quickly removed.

In this study, we demonstrated a significant reduction in the required duration of NPWT in patients treated with the HeartShield device. Although the HeartShield device was constructed for the protection of the heart and lungs, it appears to be beneficial for wound drainage. This improved drainage capacity could be responsible for accelerated wound healing, seen as a decrease in the required duration of NPWT. Despite the fact that the number of patients in this study was small, this is an interesting observation and may indicate an improvement in the treatment of patients with DSWI. This will lead to not only reduced suffering of the patient but also a reduction in health care costs.

## CONCLUSION

The use of the HeartShield device not only prevents bleeding but also appears to lead to more rapid healing of the infected thoracic cavity, possibly due to increased drainage capacity. This would reduce the duration of hospital stay for this category of patients. The authors are shareholders of ShieldHeart MedTech AB that manufactures HeartShield®.
